# Efficacy of Novel Bacteriophages against *Escherichia coli* Biofilms on Stainless Steel

**DOI:** 10.3390/antibiotics10101150

**Published:** 2021-09-24

**Authors:** Jean Pierre González-Gómez, Berenice González-Torres, Pedro Javier Guerrero-Medina, Osvaldo López-Cuevas, Cristóbal Chaidez, María Guadalupe Avila-Novoa, Melesio Gutiérrez-Lomelí

**Affiliations:** 1 Centro de Investigación en Biotecnología Microbiana y Alimentaria, Departamento de Ciencias Básicas, División de Desarrollo Biotecnológico, Centro Universitario de la Ciénega, Universidad de Guadalajara, Av. Universidad 1115, Ocotlán 47820, Mexico; jgonzalez.219@estudiantes.ciad.mx (J.P.G.-G.); berenice.gonzalez.220@estudiantes.ciad.mx (B.G.-T.); pjgm@cuci.udg.mx (P.J.G.-M.); 2 Laboratorio Nacional para la Investigación en Inocuidad Alimentaria (LANIIA), Centro de Investigación en Alimentación y Desarrollo, A.C. (CIAD), Carretera a Eldorado Km 5.5, Culiacán 80110, Mexico; osvaldo.lopez@ciad.mx (O.L.-C.); chaqui@ciad.mx (C.C.)

**Keywords:** *E. coli* biofilms, food contact surfaces, biocontrol, bacteriophages

## Abstract

Biofilm formation by *E. coli* is a serious threat to meat processing plants. Chemical disinfectants often fail to eliminate biofilms; thus, bacteriophages are a promising alternative to solve this problem, since they are widely distributed, environmentally friendly, and nontoxic to humans. In this study, the biofilm formation of 10 *E. coli* strains isolated from the meat industry and *E. coli* ATCC BAA-1430 and ATCC 11303 were evaluated. Three strains, isolated from the meat contact surfaces, showed adhesion ability and produced extracellular polymeric substances. Biofilms of these three strains were developed onto stainless steel (SS) surfaces and enumerated at 2, 12, 24, 48, and 120 h, and were visualized by scanning electron microscopy. Subsequently, three bacteriophages showing podovirus morphology were isolated from ground beef and poultry liver samples, which showed lytic activity against the abovementioned biofilm-forming strains. SS surfaces with biofilms of 2, 14, and 48 h maturity were treated with mixed and individual bacteriophages at 8 and 9 log_10_ PFU/mL for 1 h. The results showed reductions greater than 6 log_10_ CFU/cm^2^ as a result of exposing SS surfaces with biofilms of 24 h maturity to 9 log_10_ PFU/mL of bacteriophages; however, the *E. coli* and bacteriophage strains, phage concentration, and biofilm development stage had significant effects on biofilm reduction (*p* < 0.05). In conclusion, the isolated bacteriophages showed effectiveness at reducing biofilms of isolated *E. coli*; however, it is necessary to increase the libraries of phages with lytic activity against the strains isolated from production environments.

## 1. Introduction

The meat industry is generally at risk, as many foodborne pathogens such as *Escherichia coli* O157:H7, *Salmonella enterica*, and *Listeria monocytogenes* can form biofilms. The microbial complex communities known as biofilms represent a serious food safety concern [1,2]. Surfaces in meat processing plants have been recognized as an important niche for biofilm formation, and the catalytic reactions that occur during their establishment can damage them. Chemical disinfection is often ineffective for the removal of biofilms due to their matrix, mainly formed from extracellular polymeric substances (EPS), which works as a diffusion barrier, preventing sanitizers from reaching biofilm-forming bacteria [2,3].

*Escherichia coli* is an abundant bacterium in the production environment of the meat industry. It is usually harmless, although, currently, more than 250 different serogroups of Shiga toxigenic *E. coli* (STEC) have been described and over 150 of these were associated with intra- and extraintestinal consumer diseases [4,5,6]. Stainless steel (SS) is the main material used as a surface during the slaughtering and manipulation of meat. However, *E. coli* is capable of forming biofilms on it, so from these biofilms, viable pathogens could become detached and lead to cross-contamination [7,8]. Thus, due to the high *E. coli* incidence in meat processing plants and the poor accessibility to and difficulty of regular cleaning and disinfection procedures, surface biofilms may pose a food safety concern.

Biofilms play a protective role for bacteria against chemical disinfection in meat processing plants. Thus, the use of bacteriophages (phages) to eliminate biofilms is a promising approach, as they show interesting properties in terms of biofilm removal through the production of enzymes that allow them to actively penetrate and disrupt biofilms [9]. These viruses can infect bacteria following lytic or lysogenic cycles. The lytic cycle ends with the lysis of the host and the release of progeny ready to infect the surrounding bacteria [1]. The use of bacteriophages as an additive in beef and poultry products was approved by the FDA in 2006 [10], and the potential of phages as a biocontrol method became popular after this event, giving rise to multiple studies into the elimination or reduction of both the pathogenic and spoilage bacteria that can be found within the food industry in biofilms or planktonic cells [11,12,13,14].

The interaction between bacteriophages and biofilms was described even before they were approved as additives. Recent studies have reported that several factors influence biofilm reduction, such as the ability of the strain to form biofilms, the biofilm development stage, the affinity of the bacteriophages for the strains that produce the biofilm, and the concentration of the bacteriophage, as well as whether it is applied individually or as a mixture [1,15,16,17]. The aim of this study was to isolate, characterize, and challenge natural lytic bacteriophages against biofilm-forming *E. coli* strains isolated from the meat industry and to evaluate the main factors that influence their effectiveness in the removal of biofilms developed on stainless steel surfaces.

## 2. Results

### 2.1. Biofilm Formation Ability of E. coli Strains

All the *E. coli* strains, isolated from the meat industry and ATCC, produced black colonies with a dry and crystalline consistency in Congo Red Agar (CRA) and were recorded as EPS producers. Whereas in the semiquantitative adherence test, MGA-EC-25 and MGA-EC-27 showed a strong adhesion ability with the highest ODs (0.898 ± 0.113 and 0.968 ± 0.042, respectively), MGA-EC-21 and *E. coli* ATCC 11303 showed a weak adhesion ability (0.095 ± 0.021 and 0.174 ± 0.018, respectively), while the rest of the strains were recorded as having null adhesion ability (Table 1).

### 2.2. Biofilm Development Curve

**Figure 1:**
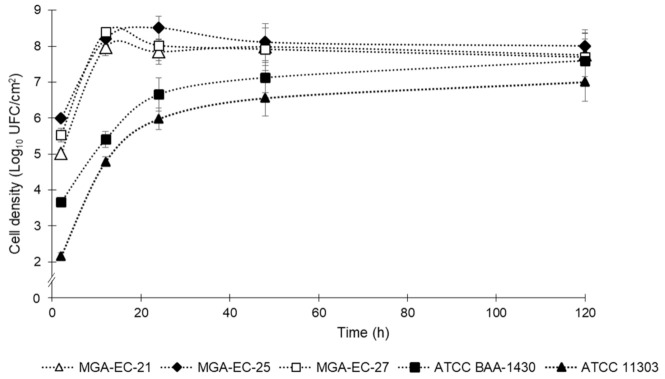
Biofilm development curve for SS coupons of the strains MGA-EC-21, MGA-EC-25, MGA-EC-27, *E. coli* ATCC BAA-1430, and *E. coli* ATCC 11303, at 2, 12, 24, 48, and 120 hours. Values are the means of three tests and vertical bars represent the standard deviations.

MGA-EC-21, MGA-EC-25, and MGA-EC-27 were selected for determination of the biofilm formation curve, the *E. coli* strain ATCC BAA-1430 was included as a surrogate indicator, and *E. coli* ATCC 11303 was included as a positive control for its reported biofilm-forming ability. The biofilms’ cell densities are summarized in Figure 1. The results showed that the three strains isolated from the meat industry reached a higher cell density in the early stages of biofilm formation compared with the ATCC; however, at 120 h, the densities reached by all the strains were between 7 and 8 log_10_ CFU/cm^2^. Moreover, in the strains from the meat industry, we observed that the highest bacterial counts were reached at 12 h, followed by the typical detachment phase of biofilms at 24 h; subsequently, the cell densities remained constant until 120 h, related to biofilm establishment. Conversely, the ATCC strains showed a constant increase in biofilm cell density at the early stages, reaching concentrations similar to those isolated from the meat industry at 120 h.

### 2.3. Bacteriophages Isolation and Characterization

**Figure 2:**
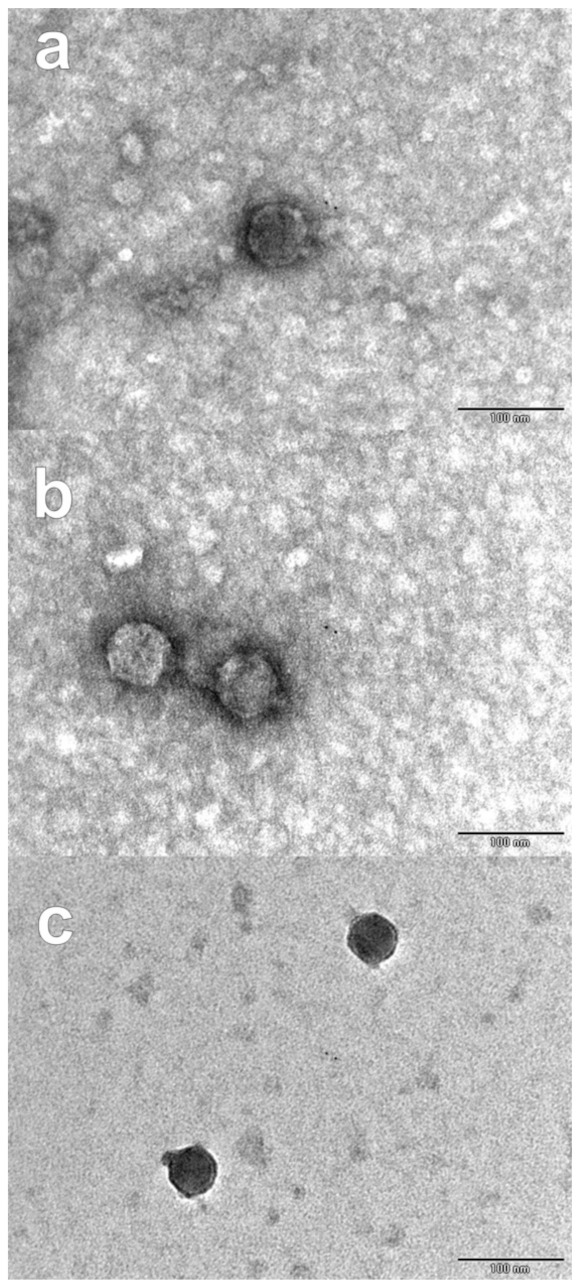
TEM micrographs of bacteriophages PL-01 (**a**), GB-02 (**b**), and GB-03 (**c**) isolated from poultry liver and ground beef.

Samples of poultry liver and ground beef were collected for the isolation of bacteriophages with lytic activity against biofilm-forming *E. coli* strains from the production environment of the meat industry. Three bacteriophages were isolated, purified, and named according to ICTV recommendations: vB_EcoP_PL-01, vB_EcoP_GB-02, and vB_EcoP_GB-03; their characterization is described in Table 2. In the host range determination, phages PL-01, GB-02, and GB-03 only produced lysis zones against the three strains from the meat industry that showed better biofilm formation ability, while none of the phages lysed any of the ATCC strains. All bacteriophages produced clear, round plaques with diameters of approximately 3 to 4 mm on the lawn of MGA-EC-27. Micrographs obtained from PL-01, GB-02, and GB-03 revealed similar podovirus morphologies with isometric heads 62.20, 51.10, and 49.39 nm in diameter, respectively, and short noncontractile tails (Figure 2).

### 2.4. Biofilm Reduction Efficacy of Bacteriophages

**Figure 3:**
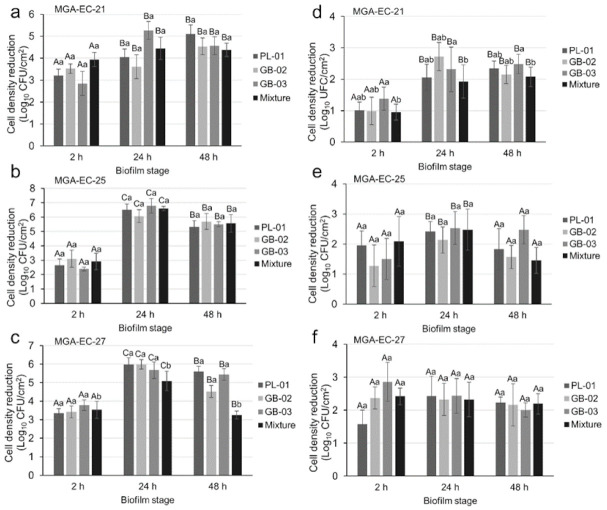
Effect of bacteriophages at concentrations of 10^9^ (**a**–**c**) and 10^8^ (**d**–**f**) PFU/mL for 1 h on MGA-EC-27, MGA-EC-25, and MGA-EC-21 biofilms at 2, 24, and 48 h of maturity. Different uppercase letters indicate a significant difference (*p* < 0.05) in the maturity of the biofilm and lowercase letters indicate a significant difference in the phage or mixture of phages applied. Values are the means of three replicates ± standard deviation.

Biofilms of three *E. coli* strains at different maturity stages were exposed to treatment with three bacteriophages individually and in a mixture at two concentrations. The results showed that depending on the *E. coli* strain, the biofilm maturity, the phages’ formulation, or the concentration applied, a statistically significant effect in reducing the cell density of the biofilm could be observed (*p* < 0.05). Afterward, an ANOVA was performed for each bacteriophage concentration because this was the factor with the greatest influence on the removal of biofilms. The treatments with bacteriophages at 10^9^ PFU/mL showed the greatest reductions, ranging from 2.39 to 6.79 log_10_ CFU/cm^2^ and reaching greater reductions at 24 and 48 h of biofilm maturity (Figure 3). Interestingly, the individual phages and the mixture had no noticeable differences in their effects. Furthermore, the reductions observed with treatments with phages at 10^8^ PFU/mL ranged between 0.95 and 2.86 log_10_ CFU/cm^2^, and the reduction effects at the different development stages were similar. The greatest reduction, 6.70 log_10_ CFU/mL, was observed when the biofilm formed by MGA-EC-25 at 24 h of maturity was exposed to phage GB-03 at a concentration of 10^9^ PFU/mL.

### 2.5. Scanning Electron Microscope Analysis

**Figure 4:**
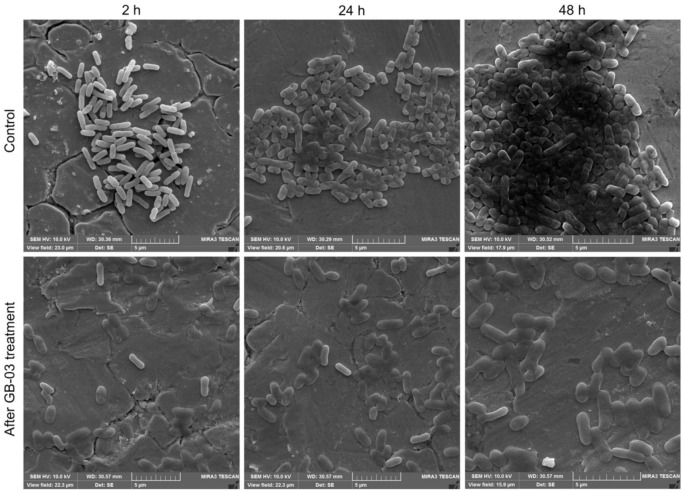
SEM micrographs of MGA-EC-25 biofilms developed on SS coupons at 2, 24, and 48 h with no treatment and after GB-03 exposure at 10^9^ PFU/mL for 1 h.

SEM micrographs showed different cell densities and morphologies at different maturity stages in *E. coli* biofilms, as summarized in Figure 4. The micrographs of the untreated surfaces showed that the biofilm density varied in the different stages of development. At 2 h of development, small groups of cells with a well-defined morphology adhering to the SS coupon were observed, while at 24 h, cell agglomerations with higher density were observed and the cell boundaries were not well defined in some regions due to the early production of extracellular polymeric substances. After 48 h of development, the biofilms showed a more compact structure in the central regions and better defined cells towards the edges; furthermore, the cells embedded in EPS formed three-dimensional structures typical of mature biofilms. Micrographs of the bacteriophage-treated surfaces showed similar results at different stages of biofilm development, with altered morphology in most of the cells due to the bacteriophages’ lytic activity and some intact cells, possibly due to the generation of phage-resistant cells or the bacteriophage failing to reach its receptor and infect the cell.

## 3. Discussion

Biofilms formed on food contact surfaces can reduce the effectiveness of sanitizers, damage the equipment, and contaminate the food product, which may lead to significant public health problems [18]. This study was conducted to evaluate the biofilm-forming ability of *E. coli* strains present in the meat industry environment and to evaluate the efficacy of novel bacteriophages as a potential biocontrol method to remove *E. coli* biofilms. Our results indicate the presence of strong biofilm-forming *E. coli* strains in the meat industry production environment. The Congo Red agar method showed that 10 (100%) *E. coli* strains from the meat industry were positive for EPS production; however, only two (20%) strains showed strong adhesion ability and one (10%) showed weak adhesion ability. The strain *E. coli* ATCC 11303, previously described as biofilm-forming [15], was also positive to EPS production and showed a weak adhesion ability. In similar studies, different proportions of EPS-producing strains and adherence ability have been found. Onmaz et al. [19] reported that 24% of *E. coli* strains isolated were EPS producers, and 36% showed at least weak adhesion ability. The majority of studies were based solely on using the adhesion ability to classify the strains as biofilm-forming [6,20,21,22]; however, we recommend performing both tests to have a better characterization of the main factors that lead the strains to produce biofilms. These studies reported results ranging from 11% to 100% of strains that showed some adhesion ability, suggesting that this characteristic is highly variable within the same species, which may be due to differences in flagellar motility, which helps the bacteria to counteract the electrostatic and hydrodynamic forces near surfaces, or it may depend on the type of surface because adhesion to abiotic surfaces is mediated by electrostatic and physicochemical interactions among the bacterial membrane, the surfaces, and the medium to which they are exposed [23,24]. Interestingly, the MGA-EC-25 and MGA-EC-27 strains showed 0.898 ± 0.113 and 0.968 ± 0.042 OD, respectively; these values are significantly higher than those for the EMC17 strain (approximately 0.43 OD) [20], the highest value reported in previous studies to our knowledge.

The *E. coli* biofilm stages on SS were observed in the development kinetics and the SEM micrographs. In the first 2 hours of development, relatively low counts were obtained in all evaluated strains, which could be due to the absence of organic matter, which enhances the initial adhesion through preconditioning the surface [25]. At 12 h, the counts increased considerably in the three strains obtained from the meat industry, reaching densities of approximately 8 log_10_ CFU/cm^2^, while both ATCC strains also increased, reaching approximately 5 log_10_ CFU/cm^2^, showing the highest point of the reversible initial adsorption. The cell desorption phase was observed at 24 h of development, when a decrease in the counts of the meat industry strains was obtained, while the ATCC strains continued slower but constant development of the biofilms. The following phases of biofilm formation (irreversible adhesion, microcolony formation, and maturation) were observed in the kinetics as stabilization of the cell density up to 128 h. These results are similar to those obtained by Carson et al. [26], who used the ATCC 11303 strain for an evaluation of biofilm development on polycarbonate, showing cell densities of 6 log_10_ CFU/well at 24 h of development, close to the 5.98 ± 0.30 log_10_ CFU/cm^2^ obtained in this study, notwithstanding that the surfaces were different. The surrogate strain ATCC BAA-1430 is recommended for the validation of disinfection methods and critical control points in production environments [27]; however, the results show that the strains from the meat industry had a greater ability to form biofilms than this surrogate; therefore, these data should be considered to choose a representative strain to evaluate disinfectants for biofilm removal. In this study, *E. coli* strains isolated from the meat industry reached considerably higher biofilm populations than those reported in similar work; however, most previous research used ATCC strains for the evaluation of biofilms, and information on strains isolated from production environments is scarce. Wang et al. [28] evaluated *E. coli* O145:H25 EC19990166 biofilms on SS surfaces; after 24 and 48 h of development, the biofilms showed populations of 4.7 ± 0.2 and 5.4 ± 0.2 log_10_ CFU/coupon (~8 cm^2^), respectively. Likewise, Kang et al. [29] evaluated the multistrain biofilm of *E. coli* O157:H7 ATCC 8624, 2026, and 2029 after 5 days of development and obtained populations of 5.90 log_10_ CFU/cm^2^. In this study, we obtained populations of approximately 8 log_10_ CFU/cm^2^ in the biofilms of three strains of *E. coli* from 24 to 120 h, with slight fluctuations, that is, 2 to 3 log_10_ more than in previous studies. Therefore, it is necessary to characterize a greater number of strains from production environments since, as our results show, their biofilm formation capacity is greater than the strains that belong to a collection, and thus, strains from production environments should be used to evaluate disinfection methods. In addition, it should be considered that in real production environments, biofilms are formed by multiple strains, species, and bacterial genera, aside from the different temperatures, humidity, organic matter, and other factors that could affect the population and composition of the biofilm [25,30].

In this study, the evaluation of three novel bacteriophages against *E. coli* biofilms was conducted. Based on the morphological properties of phages PL-01, GB-02, and GB-03, they would have been classified in the order *Caudovirales* within the family *Podoviridae* [31]. However, in recent years, the taxonomy of viruses has changed and now nine families are recognized within the *Caudovirales* order and, in addition to their morphology, their genomic characteristics must be considered for a correct classification [32]. These bacteriophages showed affinity against strains with a greater ability to form biofilms, probably due to the presence of a specific receptor, which could be verified by further studies on phage-resistant bacteria to analyze if this receptor has been lost and how this affects the ability of biofilm formation, as reduced virulence has been shown in other bacteria after losing the phage-specific receptor (capsular polysaccharides, teichoic acids, and pilus) [33]. Both individual phages and phage cocktails were challenged against biofilms of three *E. coli* strains at 2, 24, and 48 h of development, showing results ranging from 0.95 log_10_ CFU/mL to 6.70 log_10_ CFU/mL of biofilm reduction after 1 hour of treatment; greater efficacy was observed when applying the treatment at a concentration of 10^9^ PFU/mL at 24 and 48 h of biofilm development. Interestingly, the individual phages showed reductions equal or greater than the cocktail in most treatments. Similar results were reported by Montso et al. [21], who reported that the individual phages obtained greater efficacy than the cocktails after 1 day of treatment; nevertheless, the authors observed that after 7 days, there was bacterial regrowth in the treatments with individual phages but not in the cocktails. In addition to being effective at preventing bacterial growth, phage cocktails can be used to overcome the generation of phage resistance and to expand the range of bactericidal action [34,35]. Other studies have reported reductions ranging from 2.9 log_10_ CFU/coupon [28] and 3.8 log_10_ CFU/cm^2^ [36] to 4.5 log_10_ CFU/blade [7], which highlights the great efficacy of the phages isolated in this study at reducing *E. coli* biofilms, especially at 24 and 48 h of development.

Bacteriophages as a biofilm biocontrol method have great potential, but it is necessary to fully understand the phage–biofilm interaction to implement all possible improvements. The first important step for phage infection is the adsorption to its receptor, and the biofilm matrix represents a barrier between the phage and its receptor. Multiple studies have shown that some phages possess depolymerase polysaccharides in the spike or tail spicule proteins, and these enzymes allow phages to degrade the polysaccharides that form the extracellular matrix and facilitate their dispersion through the biofilm [1,37,38]. Furthermore, bacteriophages having a podovirus (short tail) morphology can diffuse better through the biofilm compared with siphoviruses and myoviruses. The enzymes present in the phages have been studied for their potential to be used individually for the removal of biofilms, since they have advantages over bacteriophages such as their greater host range, there is no risk of transferring virulence genes, and no resistant bacteria are produced [1]. The lytic activity shown by the bacteriophages PL-01, GB-02, and GB-03 specifically against the *E. coli* strains with the highest biofilm formation ability and high rates of biofilm reduction may be due to binding to a receptor that is involved in the formation of EPS and the presence of enzymes that allow the degradation of the extracellular matrix [1]. To achieve a better understanding of these interactions, it is necessary to perform the sequencing of the phages, *E. coli* strains, and phage-resistant strains to determine the specific receptor of these phages and to investigate their role in the formation of biofilms, as well as to obtain and purify the phages’ proteins to characterize their depolymerase activities.

## 4. Materials and Methods

### 4.1. Bacterial Strains

The *E. coli* strains used in the present study were isolated from the surfaces of meat processing plants. These strains were kindly provided by the Microbiology Laboratory of Cuciénega UDG (Table 1). Additionally, the surrogate indicator strain *E. coli* ATCC BAA-1430 was used as a reference strain, and *E. coli* ATCC 11303 was used as a positive control for its reported biofilm formation ability. All the strains were subcultured in tryptic soy broth (TSB; Becton Dickinson Bioxon, Le Pont de Claix, France) for 24 h at 37 °C to obtain a final concentration of 10^8^ CFU/mL.

### 4.2. Characterization of the Strains’ Biofilm-Forming Ability

#### 4.2.1. Production of Extracellular Polymeric Substances (EPS)

EPS production was evaluated according to the CRA method described by Mariana et al. [39], with some modifications. The test was carried out with two formulations: the first was prepared with a blood agar base, with 0.4 g/L Congo Red and 36 g/L glucose added; in the second formulation, glucose was replaced by 36 g/L sucrose. *E. coli* strains were inoculated in the medium and incubated under aerobic conditions for 48 h at 37 °C. The strains that produced black colonies with a dry crystalline consistency were recorded as EPS producers, while those that grew as red colonies were recorded as nonproducers.

#### 4.2.2. Semiquantitative Adherence Assay

The ability of the strains to adhere to abiotic surfaces was evaluated in 96-well flat-bottomed microtiter polystyrene plates according to the method described by Milanov et al. [40], with some modifications. For each strain, 3 wells of the microtiter plate were filled with 200 μL of a bacterial suspension in TSB with 0.5% glucose (*w/v*) (TSB + G), 3 wells were filled with the TSB + G and used as negative controls, and *E. coli* ATCC 11303 was used as the positive control. The plates were then incubated at 37 °C for 24 h. Briefly, the contents of the wells were removed by inverting the plates, and each well was washed 3 times with phosphate-buffered saline (PBS; 7 mM Na_2_HPO_4_, 3 mM NaH_2_PO_4_, and 130 mM NaCl; pH 7.4) to remove the planktonic bacteria. The attached bacteria were fixed with 100 μL of 95% ethanol for 5 min and the plates were emptied and left to dry. Staining was performed with 100 µL of 1% crystal violet for 5 min, then 3 washes with PBS were carried out, and the plates were allowed to dry at room temperature. The plates were stained with 100 µL of 1% (*w/v*) crystal violet solution per well for 5 min. The excess stain was rinsed off with sterile distilled water, and the microtiter plates were air-dried. Optical density (OD) was measured at λ = 570 nm using the Multiskan FC (Thermo Fisher Scientific Inc., Madison, WI, USA). The cutoff value of OD (ODc) was defined as three standard deviations above the mean OD of the negative control. The strains were classified as having null adherent ability (OD ≤ ODc), weak adhesion ability (ODc < OD ≤ 2 × ODc), moderate adhesion ability (2 × ODc < OD ≤ 4 × ODc), or strong adhesion ability (4 × ODc < OD).

### 4.3. Biofilm Formation on Stainless Steel

#### 4.3.1. Biofilm Quantification

The biofilm-formation ability of *E. coli* was investigated on stainless steel (SS; AISI 316, 8 × 20 × 1 mm) coupons. SS coupons, previously treated and sterilized, were placed individually into the glass test tubes (20 × 150 mm) containing 5 mL of TSB + G [41,42]. For each strain, 5 tubes were inoculated with 50 μL of the bacterial culture (10^8^ CFU/mL) and were incubated at 22 ± 2 °C for 2, 12, 24, 48, and 120 h, respectively. After incubation, SS coupons were removed under sterile conditions using sterile forceps and rinsed 2 times by pipetting 1 mL of PBS, and placed independently in tubes containing 9 mL of casein peptone (BD, Bioxon, Becton Dickinson, Le Pont de Claix, France), and the biofilms were removed by sonication (50–60 Hz for 1 min; Sonicor Model SC-100TH, West Babylon, NY, USA). Serial dilutions and conventional plating on tryptic soy agar (TSA; Becton Dickinson, Le Pont de Claix, France) were used to estimate the viable cells in the biofilm. The plates were incubated at 37 °C for 24 h. Three replicates were performed for each strain, and an SS coupon without inoculum was included in all assays as a negative control.

#### 4.3.2. Scanning Electron Microscopy

After each incubation period, the coupon was removed from the tube, rinsed with PBS, and immersed in 2% glutaraldehyde at 4 °C for 2 h to fix the adhering bacteria [43]. Briefly, the SS coupons were vacuum-dried and gold-coated for 30 s [44]. Biofilms were observed by using a TESCAN Mira3 LMU scanning electron microscope (Brno-Kohoutovice, Czech Republic).

### 4.4. Bacteriophage Isolation

Twenty samples of ground beef and poultry liver were collected from the municipal market of Ocotlán, Jalisco, Mexico. Three milliliters of an overnight culture of each of the 10 previously isolated *E. coli* strains grown in TSB was mixed with 5 g of ground beef or chicken liver in a sterile 50 mL conical tube. The enriched samples were incubated for 24 h at 37 °C and 70 rpm in a shaking bath. The tubes were centrifuged at 10,000× *g* for 10 min at 4 °C (Megafuge 16R, Thermo Fisher Scientific Inc., Waltham, MA, USA), and the supernatant was filtered twice through a sterile nitrocellulose membrane (0.45 and 0.22 µm pore diameter, respectively), using a vacuum pump. The filtered samples (lysates) were used to perform the SPOT test, through the soft overlay technique with 0.4% agarose, against 10 *E. coli* strains [45,46]. The soft agar technique, which involved mixing 1 mL of the overnight cultures with 100 μL of the filtrates that showed lytic activity, was used to observe the production of plaques. Plaques were selected on the basis of size and clarity and transferred to microtubes containing 1 mL of nanopure water. The procedure was repeated at least 3 times per sample to obtain purified phages [7,15,40,47,48].

#### 4.4.1. Phage Host Range

Ten *E. coli* strains were used to test the infection spectrum of the isolated phages. The bacterial strains were cultured in TSB at 37 °C overnight with constant shaking (70 rpm), and 1 mL of each strain was mixed with 3 mL of 0.4% top agarose at 45 °C. Briefly, the suspension was poured into a petri dish with TSA and solidified at room temperature. Next, 10 µL of each phage suspension (10^8^–10^9^ PFU/mL) was spotted on the soft agar overlay, left to dry, and incubated for 18–24 h at 37 °C [46]. The results were interpreted and recorded as follows: a clear zone of complete lysis: ++; incomplete lysis: +; no lysis: - [49].

#### 4.4.2. Phage Morphology Determined by Transmission Electron Microscopy

The 3 isolated phages were examined by transmission electron microscopy (TEM). A drop of high-titer phage stock (approximately 10^9^ PFU/mL) was placed on the surface of a formvar-coated grid (400 mesh copper grid), negatively stained with 2% phosphotungstic acid (pH 7.2) for 5 min, and the excess was removed with filter paper. The grid was carbon-shadowed in a vacuum evaporator (JEOL, JEE400). Electron micrographs were taken at various magnifications in a JEOL JEM-1011 transmission electron microscope [49].

### 4.5. Biofilm Exposure to Bacteriophages

Biofilms of the *E. coli* strains that showed greater adherence ability (MGA-EC-21, MGA-EC-25, and MGA-EC-27) were promoted on SS coupons as described in Section 4.3.1. After 2, 24, and 48 h of incubation time, the coupons were removed under sterile conditions and washed with 1 mL of PBS to remove planktonic cells. Each SS coupon was deposited in 3 mL of the bacteriophage solution (individual or mixed) at concentrations of 10^8^ or 10^9^ PFU/mL and exposed for 1 h. The coupon was then extracted from the phage solution and washed with 1 mL of PBS, and the biofilms were removed by sonication in a tube with 9 mL of casein peptone. Serial dilutions and standard plates on TSA were used to estimate viable cells in the biofilm after exposure to phages, and the results obtained were compared with the biofilm development curve of each strain to obtain the cell density reduction [7,14,30]. Three replicates were performed per treatment. 

### 4.6. Statistical Analysis

All the experiments were performed in triplicate, and the data were evaluated using analysis of variance (ANOVA), followed by a least significant difference (LDS) test, in Statgraphics Centurion XV software v15.2.06 (Statpoint Technologies, Inc., Warrenton, VA, USA).

## 5. Conclusions

The present study showed that *E. coli* strains isolated from the meat industry are capable of producing EPS, and some of them showed the ability to adhere to surfaces and produced mature biofilms on stainless steel at 48 h of development. Furthermore, the three isolated bacteriophages showed affinity against the strains with the highest biofilm formation capacity and showed efficient lytic activity against *E. coli* biofilms, mainly at 24 and 48 h of maturity. Interestingly, the application of the bacteriophages in a mixture did not show a greater efficiency compared with the application of individual phages; in some treatments, the effectiveness of the individual application was significantly higher than that of the phage mixture. Nevertheless, the application of mixtures of bacteriophages has multiple advantages, such as the wide range of strains that can be infected or the generation of bacteria that are resistant to one of the bacteriophages but can be infected by another phage in the mixture. Bacteriophages have the potential to be used as a biocontrol method against *E. coli* biofilms in the meat industry, reducing the risk of product contamination and avoiding the deterioration of equipment and surfaces. However, it is necessary to characterize the biofilm formation ability of the strains of interest and to increase the libraries of phages that show specific activity against the biofilm-producing strains, in addition to characterizing the phage enzymes that can also be used for the removal of biofilms. The enzymes present in the phages have been studied for their potential to be used individually for the removal of biofilms, since they have advantages over bacteriophages, such as the greater range of hosts, there is no risk of transferring virulence genes, and no resistant bacteria are produced.

## Figures and Tables

**Table 1:** Characterization of the biofilm formation ability of the *E. coli* strains used in this study.

Bacterial Strain	Adherence Assay		Phenotype CRA ^b^
	OD (*λ* = 570)	Adhesion Ability ^a^	
MGA-EC-01	0.076 ± 0.008	Null	EPS producer
MGA-EC-02	0.073 ± 0.008	Null	EPS producer
MGA-EC-08	0.066 ± 0.004	Null	EPS producer
MGA-EC-21	0.095 ± 0.021	Weak	EPS producer
MGA-EC-23	0.067 ± 0.005	Null	EPS producer
MGA-EC-25	0.898 ± 0.113	Strong	EPS producer
MGA-EC-26	0.093 ± 0.026	Null	EPS producer
MGA-EC-27	0.968 ± 0.042	Strong	EPS producer
MGA-EC-28	0.071 ± 0.007	Null	EPS producer
MGA-EC-30	0.062 ± 0.001	Null	EPS producer
ATCC BAA-1430	0.074 ± 0.003	Null	EPS producer
ATCC 11303	0.174 ± 0.018	Weak	EPS producer

^a^ Null adherent ability: OD ≤ 0.093; weak adhesion ability: 0.093 < OD ≤ 0.186; moderate adhesion ability: 0.186 < OD ≤ 0.373; strong adhesion ability: 0.373 < OD. ^b^ EPS producer: black colonies of dry crystalline consistency in CRA; EPS nonproducer: red colonies in CRA.

**Table 2:** Characterization of bacteriophages with lytic activity against biofilm-forming *E. coli* strains.

Phage Strain	Morphology ^a^	Source	Host Range (MGA-EC Strains) ^b^										Plaque Diameter ^c^
			01	02	08	21	23	25	26	27	28	30	
PL-01	Podovirus	Poultry liver	-	±	±	++	-	++	-	++	-	-	3 mm
GB-02	Podovirus	Ground beef	±	-	-	+	-	+	-	+	-	+	4 mm
GB-03	Podovirus	Ground beef	±	-	-	++	-	++	-	++	-	±	3.5 mm

^a^ Classification according to the morphological characteristics of TEM micrographs following the guidelines of the International Committee on Taxonomy of Viruses. ^b^ Host range results were recorded as follows: clear zone of complete lysis: ++; clear zone of lysis: +; incomplete lysis: ±; no lysis: -. ^c^ Plaque diameter obtained via the double agar technique using the MGA-EC-27 strain as the host.

## Data Availability

The data used to support the findings of this study are available from the corresponding authors upon request.
